# Causal associations between dried fruit intake and cardiovascular disease: A Mendelian randomization study

**DOI:** 10.3389/fcvm.2023.1080252

**Published:** 2023-02-06

**Authors:** Youjie Zeng, Si Cao, Heng Yang

**Affiliations:** ^1^Department of Anesthesiology, Third Xiangya Hospital, Central South University, Changsha, Hunan, China; ^2^Department of Neurology, Third Xiangya Hospital, Central South University, Changsha, Hunan, China

**Keywords:** cardiovascular disease, heart failure, coronary artery disease, myocardial infarction, ischemic stroke, causal relationship, incidence risk, single-nucleotide polymorphisms

## Abstract

**Background:**

Previous studies have shown controversy about whether dried fruit intake is associated with cardiovascular disease. This study aimed to examine the potential causal effect of dried fruit intake on cardiovascular disease by conducting a two-sample Mendelian randomization study.

**Methods:**

We used genome-wide association study (GWAS) summary statistics for MR analysis to explore the causal association of dried fruit intake with CVD. The inverse-variance weighted (IVW) method was used as the main analytical method for MR analysis. In addition, the MR-Egger method and the weighted median method were applied to supplement the IVW method. Furthermore, Cochrane’s *Q* test, MR-Egger intercept test, MR-PRESSO global test, and leave-one-out analysis were used to perform sensitivity analysis.

**Results:**

The results from the IVW analysis indicated that dried fruit intake could reduce the risk of heart failure [odds ratio (OR) = 0.6014, 95% confidence interval (CI): 0.4243–0.8522, *p*-value = 0.0043], total ischemic stroke (OR = 0.4547, 95% CI: 0.2950–0.7010, *p*-value = 0.0004), and small vessel stroke (OR = 0.3499, 95% CI: 0.1466–0.8349, *p*-value = 0.0180). In addition, the results of two additional methods (MR Egger and Weighted median) were parallel to the effects estimated by IVW. Furthermore, the sensitivity analysis illustrates that our MR analysis was unaffected by heterogeneity and horizontal pleiotropy. Finally, the results of the leave-one-out method showed the robustness of our MR results.

**Conclusion:**

Our study provides evidence for the benefits of dried fruit intake on CVD. Therefore a reasonable consumption of dried fruit may provide primary prevention.

## 1. Introduction

Cardiovascular disease (CVD) refers to a variety of heart and vascular conditions, such as coronary artery disease (CAD), cerebrovascular disease, and heart failure (HF) (1). CVD is currently the leading cause of death and disability worldwide, despite numerous advances in treatment (2). Therefore, it is necessary to find more protective factors to prevent CVD progression.

Fruit and vegetable intake is associated with the risk of cardiovascular disease, total cancer, and all-cause mortality (3). In addition, the effect of processed fruit intake on cardiovascular disease risk has also been explored in several studies. For instance, a recent meta-analysis showed a significantly reduced overall cardiovascular disease risk with 78 ml of 100% fruit juice intake per day compared to no 100% fruit juice intake (4). Another widely investigated processed fruit is dried fruit, a stable form of fruit that preserves freshness through drying techniques (5). Despite frequently serving as a daily snack, dried fruit intake can potentially benefit human health (6). An observational study suggests that dried fruit intake may potentially contribute to preventing gastrointestinal cancers (7). Raisin intake reduces plasma lipid and inflammatory cytokine levels (8). In addition, Bays et al. (9) reported that raisins reduced postprandial glucose levels and systolic blood pressure. Furthermore, dried fruits and nuts have been reported to help promote cardiometabolic health (10). Nonetheless, Sullivan VK found that dried fruit consumption did not reduce cardiometabolic risk factors (11). Therefore, these controversial findings highlight the need for further research to determine the causal relationship between dried fruit intake and cardiovascular disease. To our knowledge, no researcher has studied the causal association of dried fruit intake with CVD using the Mendelian randomization (MR) method.

Observational studies focus only on the correlation between exposure and outcome rather than concluding causal associations. In addition, observable and unobservable residual confounders may lead to biased or opposite conclusions. Similar to randomized controlled trials, the MR study is a novel research method for exploring the causal association between exposure and outcome (12). In MR studies, single nucleotide polymorphisms (SNPs) are considered instrumental variables (IVs) to estimate the causal association between exposures and the outcomes of interest (13). SNPs conform to the principle of random assignment of genetic variants at meiosis, which avoids the effect of confounding factors and the potential impact of reverse causation since genetic variants precede the onset of disease (14). A recent MR study suggests that lifestyle behaviors impact CVD risk and longevity (15). Through MR studies, more potential, influential exposure factors for CVD can be uncovered. Therefore, our study performed a two-sample MR design to investigate whether dried fruit intake is causally correlated with CVD and to provide scientific evidence for the primary prevention of CVD.

## 2. Materials and methods

### 2.1. Study design

**FIGURE 1:**
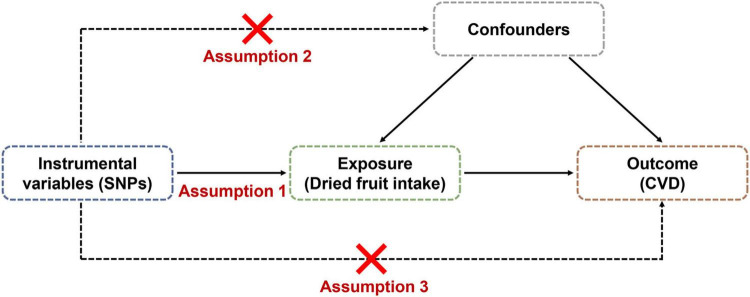
Flow diagram for Mendelian randomization (MR) study.

The flow diagram for the whole study is shown in Figure 1. MR studies are required to satisfy the following three assumptions: (i) IVs are strongly associated with exposure factors, (ii) IVs are independent of confounding factors, and (iii) IVs are solely associated with outcomes through exposure factors (13). In our study, dried fruit intake was the exposure factor, and the outcome was CVD, including coronary artery disease (CAD), myocardial infarction (MI), heart failure (HF), ischemic stroke (IS), and its subtypes. In addition, fasting glucose, fasting insulin, type 2 diabetes, body mass index (BMI), systolic blood pressure (SBP), diastolic blood pressure (DBP), low-density lipoprotein (LDL), high-density lipoprotein (HDL) and total cholesterol (TC) were considered as confounding factors.

### 2.2. Data sources for dried fruit intake exposure

The Genome-Wide Association Study (GWAS) data for dried fruit intake were derived from a large cohort study involving approximately 500,000 individuals conducted by the UK Biobank.^1^ The study collected genotypic and various phenotypic data and was approved by the research ethics committee. Participants in the cohort were invited to the local evaluation center for data collection using a touch-screen questionnaire or standardized anthropometry. Participants’ intake of dried fruits as an exposure factor was extracted by a questionnaire asking about the frequency of dried fruit intake. The participants were asked, “How many pieces of dried fruit would you eat per day?” (One prune, one dried apricot, and ten raisins are considered as one piece). In addition, three additional options, (i) less than one, (ii) do not know, and (iii) prefer not to answer, were available for participants to select. In total, 421,764 European participants’ dried fruit intake data were obtained. The GWAS summary statistics have been included in the IEU OpenGWAS database and are easily available for researchers to download (accession number: ukb-b-16576) (16, 17).

### 2.3. Data sources for CVD outcomes

Genome-wide association study summary statistics for CAD and MI were derived from the CARDIoGRAMplusC4D consortium’s study of 60,801 cases (about 70% of cases reported a history of MI) and 123,504 controls [The majority (77%) of the participants were of European ancestry] (18). GWAS summary statistics for HF were obtained from the HERMES consortium’s study that included 47,309 cases and 930,014 controls of European ancestry (19). GWAS summary statistics for any type of IS (AIS) were derived from the MEGASTROKE consortium’s study, enrolling 438,847 European participants (34,217 IS cases and 404,630 controls) (20). The case group was further subdivided into cardioembolic stroke (CES), small vessel stroke (SVS), and large artery stroke (LAS) according to the TOAST classification (21).

### 2.4. Instrumental variable selection

To identify SNPs significantly associated with dried fruit intake as valid IVs, we chose a cutoff *p*-value < 5 × 10^–8^ as genome-wide significance. In addition, SNPs within a window size of 10000 kb at a threshold of r2 < 0.001 were pruned to mitigate linkage disequilibrium (LD). In addition, we downloaded GWAS summary data of fasting glucose, fasting insulin, type 2 diabetes, BMI, SBP, DBP, LDL, HDL, and TC, removing SNPs from IVs that were closely associated (*p*-value < 5 × 10^–8^) with these confounders. Sources of the GWAS summary statistics for the confounders are shown in Supplementary Table 1. Finally, we calculated the F-statistic to assess the degree of weak instrumental bias (the calculation formula is available in Supplementary Table 2). If the F statistic > 10, it was considered that no bias was caused by weak IVs (22).

### 2.5. Statistical methods

The inverse-variance weighted (IVW) method was used as the main analytical method for estimating potential causal effects, which is an extension of the Wald ratio estimator based on the principles of Meta-analysis (23). In addition, the MR-Egger method and the weighted median method were applied to supplement the IVW method (24, 25). These three approaches are considered the most scientific and commonly used methods, providing robust analysis for MR studies (26). The criterion for using the weighted median method is that at least 50% of the SNPs must satisfy the premise that they are valid IVs (25). The MR-Egger method provides unbiased estimates even when all selected IVs are multivariate (24). Results of causal associations were presented as odds ratios (OR) and 95% confidence intervals (95% CI). Cochrane’s *Q* values were used to assess heterogeneity. MR-Egger intercept test and MR-PRESSO global test were utilized to detect horizontal pleiotropy (27, 28). In addition, the leave-one-out analysis was performed to assess the robustness of the results.

## 3. Results

### 3.1. Details of IVs

We first obtained 43 SNPs that were independent of each other and strongly associated with dried fruit intake. After excluding SNPs associated with the confounding factors, 28 SNPs were finally included as IVs. Details of the 28 IVs are shown in Supplementary Table 2. The F-statistic for the 28 IVs was 14.63; thus, it can be assumed that they have a solid potential to predict the dried fruit intake level. In addition, the association of all IVs with dried fruit intake exposure was more significant than the association with CVD outcomes (Supplementary Table 3).

### 3.2. Causal effects of dried fruit intake on CVD

**FIGURE 2:**
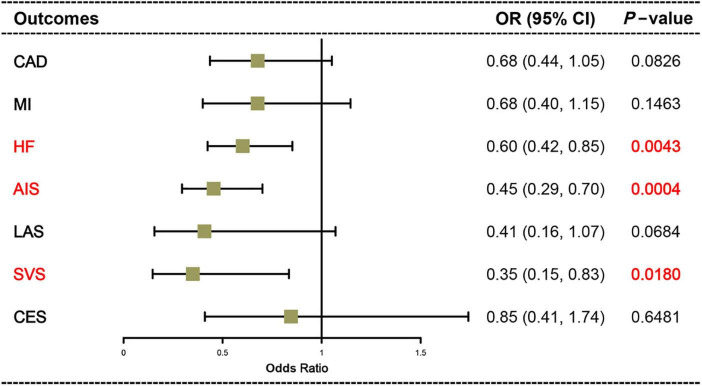
Causal effects of dried fruit intake on cardiovascular disease (CVD) assessed by the inverse-variance weighted (IVW) method. CAD, coronary artery disease; MI, myocardial infarction; HF, heart failure; AIS, any ischemic stroke; LAS, large artery stroke; SVS, small vessel stroke; CES, cardioembolic stroke.

**FIGURE 3:**
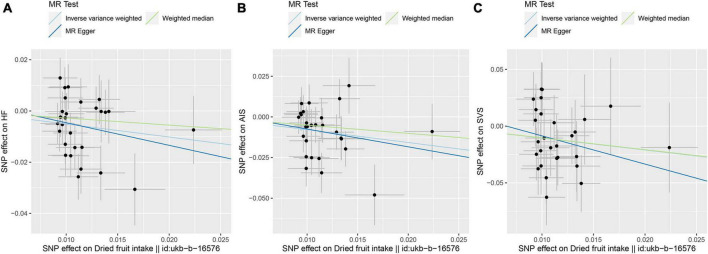
Scatter plot of Mendelian randomization (MR) results. **(A)** Scatter plot of genetic correlations of dried fruit intake and heart failure (HF) using different MR methods. **(B)** Scatter plot of genetic correlations of dried fruit intake and any ischemic stroke (AIS) using different MR methods. **(C)** Scatter plot of genetic correlations of dried fruit intake and small vessel stroke (SVS) using different MR methods.

Mendelian randomization results from the IVW method suggest a causal association of dried fruit intake with HF, AIS, and SVS (Figure 2). The higher the intake of dried fruits, the lower the risk of HF, AIS, and SVS. The risk of HF decreased by 39.86% (OR = 0.6014, 95% CI: 0.4243–0.8522, *p*-value = 0.0043) for every increase of dried fruit intake by one standard deviation (1.81836 pieces a day); the risk of AIS was reduced by 54.53% (OR = 0.4547, 95% CI: 0.2950–0.7010, *p*-value = 0.0004); the risk of SVS was reduced by 65.01% (OR = 0.3499, 95% CI: 0.1466–0.8349, *p*-value = 0.0180). Subsequently, two additional methods, MR Egger and Weighted median, were used to assess the causal association of dried fruit intake with HF, AIS, and SVS, and the results were parallel to the effects estimated by IVW (OR < 1) (Table 1 and Figure 3).

**TABLE 1:** Causal effects of dried fruit intake on HF, AIS, and SVS evaluated by IVW method, MR Egger method, and weighted median method.

Outcomes	Methods	OR (95% CI)	*P*-value
HF	IVW	0.6014 (0.4243, 0.8522)	0.0043
	MR Egger	0.4131 (0.0634, 2.6905)	0.3636
	Weighted median	0.7590 (0.4841, 1.1899)	0.2293
AIS	IVW	0.4547 (0.2950, 0.7010)	0.0004
	MR Egger	0.3347 (0.0322, 3.4754)	0.3677
	Weighted median	0.6020 (0.3406, 1.0643)	0.0809
SVS	IVW	0.3499 (0.1466, 0.8349)	0.0180
	MR Egger	0.0830 (0.0008, 9.1473)	0.3091
	Weighted median	0.3540 (0.1023, 1.2244)	0.1009

HF, heart failure; AIS, any ischemic stroke; SVS, small vessel stroke; IVW, inverse-variance weighted.

### 3.3. Sensitivity analysis

**FIGURE 4:**
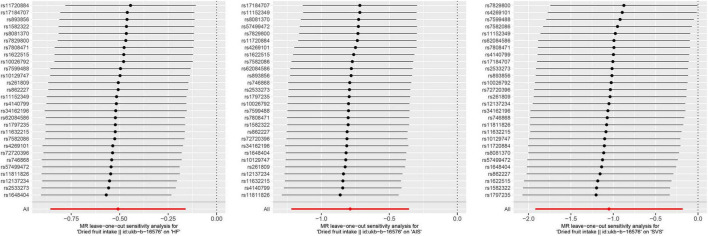
Leave-one-out analysis for dried fruit intake on heart failure (HF), any ischemic stroke (AIS), and small vessel stroke (SVS).

Cochran’s *Q*-test indicates no significant heterogeneity among the 28 IVs. [(i) HF: Q_pval_IVW_ = 0.067, Q_pval_MR–Egger_ = 0.054; (ii) AIS: Q_pval_IVW_ = 0.102, Q_pval_MR–Egger_ = 0.082; (iii) SVS: Q_pval_IVW_ = 0.410, Q_pval_MR–Egger_ = 0.377] (Table 2). In addition, the results of both the MR-Egger intercept test and MR-PRESSO global test suggested that there was no horizontal pleiotropy between dried fruit intake and HF, AIS, and SVS in our study (MR-Egger intercept test: (i) HF: intercept = 4.26E-03, *p*-value = 0.692; (ii) AIS: intercept = 3.48E-03, *p*-value = 0.796; (iii) SVS: intercept = 1.63E-02, *p*-value = 0.547. MR-PRESSO global test: (i) HF: RSS obs = 41.525, *p*-value = 0.076; (ii) AIS: RSS obs = 39.134, *p*-value = 0.121; (iii) SVS: RSS obs = 30.119, *p*-value = 0.426) (Table 3). Moreover, the leave-one-out analysis demonstrated the stability of the MR results in our study since excluding any one IV did not shift the overall results (Figure 4).

**TABLE 2:** Results of heterogeneity by Cochran’s *Q* test.

Outcome	Method	Cochran’s *Q* test		
		**Q**	**Q_df**	**Q_pval**
HF	IVW	38.736	27	0.067
	MR-Egger	38.499	26	0.054
AIS	IVW	36.627	27	0.102
	MR-Egger	36.531	26	0.082
SVS	IVW	28.023	27	0.410
	MR-Egger	27.627	26	0.377

HF, heart failure; AIS, any ischemic stroke; SVS, small vessel stroke; IVW, inverse-variance weighted.

**TABLE 3:** Results of horizontal pleiotropy by MR-Egger intercept test and MR-PRESSO global test.

Outcome	MR-egger intercept test			MR-PRESSO global test	
	**Intercept**	**SE**	***P*** **-value**	**RSS obs**	***P*** **-value**
HF	4.26E-03	0.01	0.692	41.525	0.076
AIS	3.48E-03	0.01	0.796	39.134	0.121
SVS	1.63E-02	0.03	0.547	30.119	0.426

HF, heart failure; AIS, any ischemic stroke; SVS, small vessel stroke; SE, standard error; RSS, residual sum of squares.

## 4. Discussion

This study analyzed the causal association between dried fruit intake and CVD using the two-sample MR method. The results indicated a causal association between dried fruit intake and AIS, SVS, and HF. However, no causal associations were observed with CAD, MI, LAS, and CES.

Only a few studies have reported the association between dried fruit intake and CVD. Some studies have reported that raisin intake can reduce CVD risk factors such as plasma lipids, inflammatory cytokine levels, postprandial glucose levels, and systolic blood pressure (8, 9). However, a recent study showed that dried fruit consumption did not improve cardiometabolic risk factors (11). Therefore, whether dried fruit intake can actually decrease CVD risk is inconclusive. Our study is the first known MR study to assess the causal effect of dried fruit intake on CVD.

Traditional dried fruits are high in fiber, low in fat, and a concentrated source of various micronutrients. In addition, it is more convenient and has longer shelf life compared to fresh fruit (29). Several studies have shed light on the possible protective effect of dried fruits against CVD. (i) A secondary analysis of the National Health and Nutrition Examination Survey revealed that dried fruit consumption was associated with improved nutrient intakes, higher overall diet quality scores, and lower BMI (30). Obesity is widely known to be one of the most common risk factors for CVD (31). Previous studies have revealed that a decrease in diet quality scores leads to an increased risk of CVD (32). (ii) An animal study uncovered the cholesterol-lowering and vasoprotective effects of ethanolic extract from the dried fruit of Crataegus pinnatifida (33). (iii) Some ingredients in dried fruit may play a key role in improving CVD risk. For instance, many fruits contain flavonoids, which scavenge free radicals, improve glucose tolerance and insulin sensitivity, modulate lipid metabolism and adipocyte differentiation, suppress inflammation and apoptosis, and improve endothelial dysfunction, thus reducing CVD risk (34). Several studies have reported the beneficial therapeutic effects of flavonoids in diabetic cardiovascular complications (35). Flavonoid has been demonstrated to exert a cardioprotective effect in ischemic heart disease in diabetic rats by activating PPARγ signaling and reducing left ventricular end-diastolic pressure and mean arterial pressure in diabetic rats (36). In addition, flavonoid has been recently reported to inhibit inflammation and fibrosis in the heart of STZ-induced diabetic rats by suppressing the NF-κB signaling pathway (37). (iv) Many dried fruits are rich in antioxidant vitamins, such as vitamin A, vitamin C, or vitamin E, which reduce CVD risk through interacting with free radicals and preventing oxidative damage to macromolecules such as low-density lipoprotein (38). (v) The dietary fiber in dried fruit can regulate gut microbes which contribute to reducing CVD risk by impacting a range of host physiological processes, including lipid and glucose metabolism and immune homeostasis (39).

Our study indicated that dried fruit intake significantly reduced the risk of HF, AIS, and SVS, while the improvement in CAD, MI, LAS, and CES were relatively insignificant. The potential underlying reason for the discrepancy might be that dried fruit has a more protective effect on the heart muscle than blood vessels. Specifically, the antioxidant and anti-inflammatory components of dried fruit may improve myocardial contractility by reducing oxidative stress and improving calcium handling, reducing HF risk (40). In contrast, we speculate that the beneficial components in dried fruit provide a relatively weak improvement in blood vessels. Therefore, it could only have a protective effect on SVS triggered by small vessel involvement. Likewise, it cannot provide significant protection against CAD, MI, LAS, and CES, which are triggered by insufficient perfusion due to vascular occlusion. Another potential reason for the difference in results could be that one of the main contributors of dried fruits to reduce CVD risk is to improve glucose tolerance and insulin sensitivity and then to regulate lipid and glucose metabolism, which requires a long-term process (41). Thus dried fruit was more protective against chronic diseases with less severe lesions. Overall, the protective effect of dried fruits varies for different CVDs, which requires further investigations in the future.

Our study strictly followed the three main assumptions of the MR study. For assumption 1, we adopted a strict threshold of *p*-value < 5 × 10^–8^ to screen SNPs associated with dried fruit intake as IVs. In addition, we eliminated the linkage disequilibrium of IVs. Furthermore, the F-statistic of IVs is greater than 10. For assumption 2, we downloaded GWAS summary statistics of confounders (common CVD risk factors) and excluded SNPs from the IVs that were strongly associated with these confounders (*p*-value < 5 × 10^–8^). Finally, for assumption 3, all IVs were more strongly correlated with dried fruit exposure factors than with CVD outcomes. In addition, the MR-Egger intercept test and MR-PRESSO global test suggested that the results were not influenced by horizontal pleiotropy (*p*-value > 0.05).

However, the present study has some limitations: (i) This study included individuals of essentially European ancestry, so extrapolating the findings to other populations is limiting. (ii) The specific underlying mechanisms of dried fruit effects are not fully understood. (iii) The data for dried fruit intake were derived from the UK Biobank questionnaire, and, therefore might be influenced by potential misclassification bias. Nevertheless, due to the large sample size, the bias would be mitigated to some extent. (iv) It is not easy to demonstrate that the results are entirely independent of the horizontal pleiotropy effect; nevertheless, we performed many sensitivity analyses to demonstrate the stability of the results. Overall, our study suggested that dried fruit intake may reduce the risk of ischemic stroke, especially small vessel stroke, and decrease the risk of heart failure.

## 5. Conclusion

Our study identified that dried fruit intake reduces the risk of HF, AIS, and SVS through MR analysis. The results confirmed the potential benefits of dried fruit and provided some insights into daily primary prevention measures for CVD.

## Data availability statement

Publicly available datasets were analyzed in this study. This data can be found here: (i) IEU Open GWAS project (https://gwas.mrcieu.ac.uk/); (ii) CARDIoGRAMplusC4D (http://www.cardiogramplusc4d.org/); (iii) HERMES (https://www.hermesconsortium.org/); and (iv) MEGASTROKE (https://www.megastroke.org/).

## Ethics statement

The studies involving human participants were reviewed and approved by Local Ethics Committees of consortia in the respective studies. The patients/participants provided their written informed consent to participate in this study.

## Author contributions

YZ designed the study, analyzed the data, and wrote the manuscript. SC assisted in analyzing the data and revising the manuscript. HY critically read and edited the manuscript. All authors contributed to the article and approved the submitted version.

## Footnotes

1
http://www.nealelab.is/uk-biobank/
